# Comparative Efficacy and Safety of Clevidipine Versus Nicardipine in Hypertensive Emergencies: A Systematic Review and Meta‐Analysis

**DOI:** 10.1002/clc.70419

**Published:** 2026-07-16

**Authors:** Tahir Ullah, Hamed Khan, Anfal Khan, Syed Asad Ullah Agha, Aleena Amir Malik, Aieman Naeem, Hamza Nasir, Abdul Qayum Khan, Muhammad Zaib, Kashif Yousaf

**Affiliations:** ^1^ Saidu Medical College Swat Pakistan; ^2^ Rawalpindi Medical University Rawalpindi Pakistan; ^3^ United Medical and Dental College Karachi Pakistan; ^4^ Department of Medicine, Jalal‐Abad State University named after B. Osmonov, Medical Faculty Jalal‐Abad Kyrgyzstan

**Keywords:** clevidipine, hypertensive emergency, meta‐analysis, nicardipine, systematic review

## Abstract

**Background:**

Hypertensive emergencies require prompt blood pressure reduction to prevent acute target‐organ damage. Clevidipine and nicardipine are commonly used intravenous dihydropyridine calcium channel blockers, but their comparative efficacy and safety remain uncertain. We performed a systematic review and meta‐analysis comparing clinical outcomes associated with clevidipine versus nicardipine.

**Methods:**

PubMed, the Cochrane Library, and ClinicalTrials.gov were searched from inception through July 3, 2026. Comparative studies evaluating intravenous clevidipine and nicardipine in adults requiring acute systolic blood pressure (SBP) control were included. Primary outcomes were time to target SBP and percentage of time within the target SBP range. Secondary outcomes included intensive care unit (ICU) and hospital length of stay, infusion drug volume, hypotension, tachycardia, acute kidney injury, rescue antihypertensive therapy, and in‐hospital mortality.

**Results:**

Nine studies involving 1378 patients (eight retrospective cohort studies and one randomized controlled trial) were included. No significant differences were observed between clevidipine and nicardipine in time to target SBP, percentage of time within the target SBP range, ICU or hospital length of stay, hypotension, tachycardia, acute kidney injury, rescue antihypertensive therapy, or in‐hospital mortality. Subgroup analyses demonstrated significantly faster target SBP attainment with clevidipine in stroke/neurocritical care and general hypertensive crisis populations. Clevidipine was associated with significantly lower infusion drug volume.

**Conclusions:**

Clevidipine and nicardipine demonstrate comparable efficacy and safety for hypertensive emergencies. Although clevidipine achieved lower infusion volumes and faster target SBP attainment in subgroup analyses, no consistent overall clinical superiority was observed. Larger randomized trials are needed to confirm these findings.

## Graphical abstract

Clevidipine and nicardipine demonstrated comparable efficacy and safety for hypertensive emergencies. Clevidipine required lower infusion drug volumes, while subgroup analyses suggested faster attainment of target systolic blood pressure in selected clinical settings.

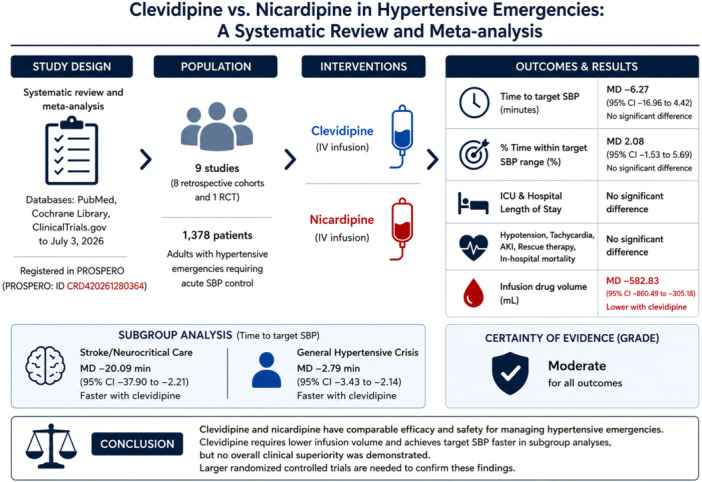

## 1 Introduction

Hypertensive emergencies are defined by accelerated and severe elevations of blood pressure associated with acute organ injury and have high morbidity. While no uniform threshold exists, blood pressure elevations typically exceed 180 mm Hg systolic and/or 110 mm Hg diastolic [1].

The 30‐day mortality rate in ICH ranges from 35% to 52%, with only 20% of survivors expected to have full functional recovery at 6 months [2]. For subarachnoid hemorrhage, a 2001 study published in Stroke, evaluating rates of rebleeding, found a significant decrease in subarachnoid hemorrhage when targeting an SBP of 160 mmHg and below [3]. In acute ischemic stroke, an SBP target of less than 180 mmHg is established to ensure thrombolytic eligibility based on the Neurological Disorders and Stroke rt‐PA Stroke Study Group trial from 1995 [4]. Nicardipine and clevidipine are both first‐line intravenous (IV) dihydropyridine (DHP) calcium channel blockers (CCB) used for rapid blood pressure control [5]. Elevated BP is thought to worsen cerebral edema as well as contribute to the risk of symptomatic ICH in patients with ischemic stroke receiving thrombolytics [6]. The pharmacokinetic and pharmacodynamic pro les of clevidipine are characterized by their rapid onset of action, linear dose‐response, steady‐state of arterial and venous levels and a peak pharmacodynamic BP response of 2 and 10 min after infusion initiation [7, 8, 9]. The American Heart Association/American Stroke Association (AHA/ASA) 2015 guidelines for spontaneous ICH stated that acute lowering of SBP to 140 mmHg is safe and can be effective for improving functional outcome [7]. Approximately one third of aneurysmal rebleeds occur within 3 h, and almost half within 6 h, of SAH onset [8]. Aneurysmal rebleeding in SAH has been associated with high morbidity and mortality; therefore, rapid control of SBP to reduce the risk of rebleeding remains essential. In the AHA/ASA 2012 guidelines for aneurysmal SAH, recommendations to prevent rebleeding included an SBP < 160 mmHg as reasonable [8].

Several critical gaps in the literature justify a systematic review and meta‐analysis. First, while individual randomized controlled trials (RCTs) have compared these agents, they are often underpowered to detect differences in clinically meaningful endpoints, such as attainment of guideline‐recommended SBP targets (e.g., SBP < 140 mmHg or < 160 mmHg) within the crucial first hour, or the incidence of overshoot hypotension. Second, the risk‐benefit trade‐off in specific cerebrovascular emergencies (ICH, SAH, acute ischemic stroke requiring thrombolysis) is not well‐defined; rapid BP lowering may worsen cerebral ischemia if too aggressive, yet delayed control increases rebleeding risk (nearly half of aneurysmal rebleeds occur within 6 h). Third, there is no current synthesis of comparative safety data regarding drug‐related adverse events (e.g., reflex tachycardia, hypotension, or the logistical burden of lipid emulsion from clevidipine). Given the high 30‐day mortality (35%−52% in ICH) and poor functional outcomes in these emergencies, even modest differences in the speed, precision, and safety of BP control could translate into significant clinical benefits.

## 2 Methods

### 2.1 Study Design

This meta‐analysis was conducted in accordance with the Preferred Reporting Items for Systematic Reviews and Meta‐Analyses (PRISMA) guidelines [10]. As the analysis was based on de‐identified retrospective clinical data and previously published studies, Institutional Review Board approval was not required. The study protocol was registered in the International Prospective Register of Systematic Reviews (PROSPERO: ID CRD420261280364).

### 2.2 Search Strategy

A structured literature search was conducted in PubMed, the Cochrane Library, and ClinicalTrials.gov from database inception through July 3, 2026, with no language restrictions applied. The complete search strategies for all databases are provided in Supporting Information S1: Appendix Table 1.

The search strategy combined Medical Subject Headings (MeSH), where applicable, and free‐text terms using Boolean operators. The PubMed search strategy included: ((“Hypertension” [MeSH Terms] OR “hypertension” [All Fields] OR “hypertensive” [All Fields] OR “hypertensive emergency” [All Fields] OR “hypertensive emergencies” [All Fields] OR “hypertensive crisis” [All Fields] OR “hypertensive crises” [All Fields]) AND (“clevidipine” [All Fields] OR “nicardipine” [All Fields])). Equivalent search strategies were adapted for the Cochrane Library and ClinicalTrials.gov.In addition to the electronic database search, the reference lists of all eligible studies and relevant review articles were manually screened to identify any additional potentially eligible studies. Titles and abstracts of the retrieved records were subsequently screened according to the predefined eligibility criteria.

### 2.3 Study Selection

Titles and abstracts were independently screened for relevance, followed by full‐text assessment of potentially eligible studies. Studies were included if they met the following criteria: (a) comparative study design (retrospective cohort, matched or paired analysis, or RCTs); (b) adult patients requiring IV antihypertensive infusion for acute systolic blood pressure (SBP) control; (c) clevidipine as the primary intervention; (d) a comparator IV antihypertensive agent (most commonly nicardipine); (e) reported quantitative outcomes related to SBP control; and (f) reported safety outcomes during infusion or hospitalization.

Exclusion criteria included: (a) non‐comparative studies, case reports, editorials, letters, or narrative reviews; (b) pediatric or animal studies; (c) conference abstracts without sufficient outcome data; and (d) studies lacking clearly defined SBP targets or quantitative blood pressure‐related outcomes.

Any disagreements during study selection were resolved through discussion with a third reviewer until consensus was reached.

### 2.4 Data Extraction

Data were extracted using a standardized data collection form. Extracted variables included: (a) study characteristics (author, year, study design, and clinical setting); (b) patient population and sample size; (c) antihypertensive intervention and comparator details; (d) SBP targets and infusion protocols; (e) follow‐up duration; (f) primary and secondary efficacy outcomes; and (g) safety outcomes, including hypotension and organ‐related adverse events.

Outcome data were extracted as reported by the original investigators. Continuous outcomes reported as means and standard deviations were extracted directly. For studies reporting continuous outcomes as medians with interquartile ranges or ranges, values were converted to estimated means and standard deviations using the method described by Wan et al. implemented through MetaConverter, prior to quantitative synthesis [20, 21]. Dichotomous outcomes were extracted as event counts. When multiple outcomes were reported, all clinically relevant measures were extracted.

### 2.5 Outcomes

Primary Outcomes: (a) Time to target SBP, defined as the elapsed time from initiation of IV antihypertensive infusion to the first documented SBP within the predefined target range specified in each study. (b) Percentage of time within the target SBP range, defined as the proportion of the treatment period during which the patient's SBP remained within the prespecified target range.

Secondary Outcomes: (a) Treatment characteristics: infusion duration, infusion drug volume, need for rescue/additional antihypertensive therapy, and transition to oral antihypertensive therapy. (b) Clinical outcomes: intensive care unit (ICU) length of stay and hospital length of stay. (c) Safety outcomes: incidence of hypotension (reported either according to the study‐specific definition or as SBP < 90 mmHg where explicitly defined), tachycardia, acute kidney injury, rebound hypertension, and in‐hospital mortality.

Study‐defined hypotension was defined as any episode of SBP falling below the predefined threshold specified in the individual study that required clinical intervention (e.g., discontinuation of the infusion, fluid administration, or vasopressor initiation). Tachycardia was defined according to the criteria reported in the individual studies. The need for rescue/additional antihypertensive therapy was defined as the requirement for an additional IV antihypertensive agent (e.g., labetalol or hydralazine) during the infusion period to achieve or maintain the target blood pressure.

Follow‐up durations ranged from the infusion period through hospital discharge, with selected studies reporting outcomes up to 30 days.

### 2.6 Risk of Bias and Study Quality Assessment

We assessed the quality of the eight observational cohort studies using the Newcastle–Ottawa Scale (NOS), which examines three main domains: selection of participants, comparability of study groups, and assessment of outcomes [22] (Supporting Information S1: Table 2). Overall, study quality ranged from moderate to high, with scores between 5 and 8 stars out of a possible 9. Three studies achieved 8 stars, four studies scored 7 stars, and one study received 5 stars.

Most studies demonstrated appropriate participant selection and clear exposure definition. However, none of the studies received a star for the fourth selection item (S4), which assesses whether the outcome of interest was absent at the start of the study. This likely reflects limitations in baseline reporting or the inherent nature of retrospective study designs, in which the initial outcome status could not always be clearly established. In terms of comparability, the majority of studies adjusted for key confounding variables, while three studies achieved full comparability by controlling for multiple important confounders. Outcome assessment was generally robust, with most studies reporting clearly defined outcomes and adequate follow‐up; minor limitations were observed in one study. Although minor methodological limitations were identified, particularly related to potential residual confounding and selection bias, the overall methodological quality of the observational studies was considered acceptable. Therefore, the findings of the meta‐analysis should be interpreted with appropriate caution; however, the generally favorable NOS ratings suggest that the likelihood of substantial bias affecting the pooled estimates is relatively low.

**Figure 1:**
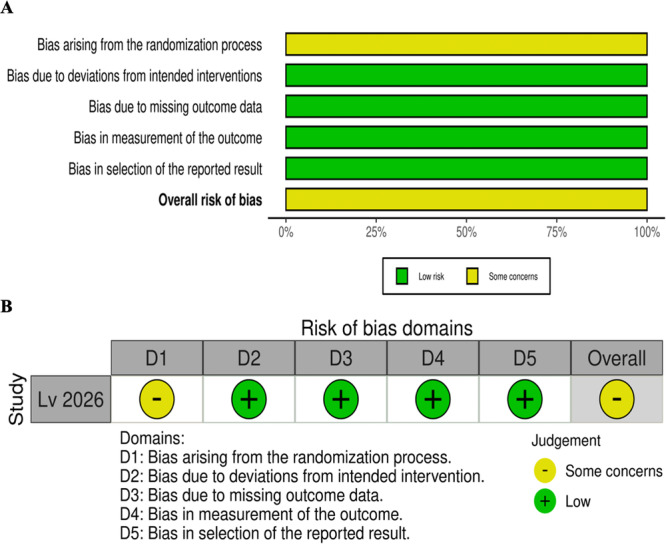
(A) Risk‐of‐bias panel a summary bar chart of included RCT (B) Risk‐of‐bias panel B Traffic light plot OF included RCT.

The single RCT [16] was assessed using the Cochrane Risk of Bias 2 (RoB 2) tool, with the results presented as a traffic light plot and summary bar chart (Figure 1) [23]. The study demonstrated a low risk of bias in four domains, including deviations from intended interventions, missing outcome data, outcome measurement, and selection of the reported result. Some concerns were identified in the randomization process domain, resulting in an overall judgment of “some concerns.” Overall, these findings indicate that the randomized trial was methodologically robust, with only minor concerns related to the randomization process, and that the overall risk of bias across the included studies was acceptable for inclusion in the quantitative synthesis.

### 2.7 Statistical Analysis

Meta‐analyses were performed using a random‐effects model because clinical and methodological heterogeneity among the included studies was anticipated. Mean differences (MDs) with 95% confidence intervals (CIs) were calculated for continuous outcomes, whereas risk ratios (RRs) with 95% CIs were calculated for dichotomous outcomes. Statistical heterogeneity was assessed using the *I* ^2^ statistic, with values of 25%, 50%, and 75% representing low, moderate, and high heterogeneity, respectively. A two‐sided *p*‐value < 0.05 was considered statistically significant. All statistical analyses were performed using Review Manager (RevMan), version 5.4 (The Cochrane Collaboration, Copenhagen, Denmark).

Formal assessment of publication bias was not performed because none of the pooled outcomes included 10 or more studies, limiting the reliability of funnel plots and statistical tests such as Egger's regression. Sensitivity analyses were not performed because the limited number of studies contributing to each outcome precluded meaningful sensitivity analyses. Subgroup comparisons were evaluated narratively based on clinical setting (neurocritical care vs. general hypertensive crisis) and study design (matched vs. unmatched cohorts) where data were available.

### 2.8 GRADE Assessment

The certainty of evidence for each outcome was evaluated using the GRADE approach [24]. Five domains were assessed: risk of bias, inconsistency, indirectness, imprecision, and other considerations. Evidence from RCTs was initially rated as high certainty, whereas evidence from observational studies was initially rated as low certainty and subsequently downgraded or upgraded according to GRADE guidance. The overall certainty of evidence was categorized as high, moderate, low, or very low. A Summary of Findings table was generated to present pooled effect estimates and certainty ratings for key outcomes (Supporting Information S1: Appendix Table 3).

## 3 Results

### 3.1 Study Selection

**Figure 2:**
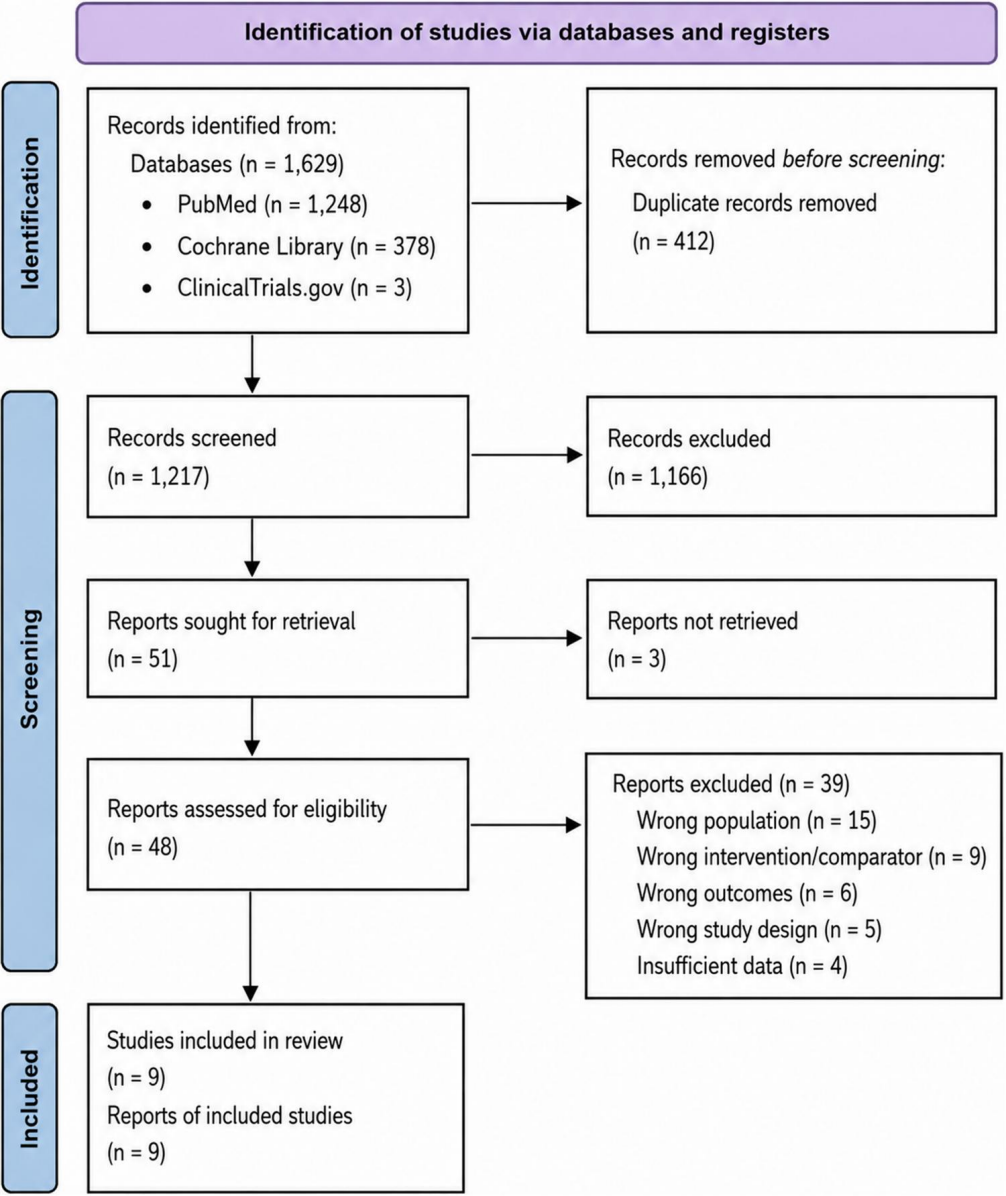
Prisma.

The literature search identified 1629 records, including 1248 from PubMed, 378 from the Cochrane Library, and 3 from ClinicalTrials.gov. After the removal of 412 duplicate records, 1217 records underwent title and abstract screening. Following screening, 1166 records were excluded, and 51 full‐text articles were sought for retrieval. Of these, three reports could not be retrieved, leaving 48 full‐text articles assessed for eligibility. After full‐text review, 39 articles were excluded for not meeting the predefined eligibility criteria, resulting in nine studies being included in the systematic review and quantitative meta‐analysis (Figure 2) [11, 12, 13, 14, 15, 16, 17, 18, 19].

### 3.2 Description of Included Studies and Patient Characteristics

A total of nine studies comparing clevidipine and nicardipine for the management of acute hypertension were included in the systematic review and meta‐analysis, encompassing 1378 patients. The included studies comprised eight retrospective cohort studies and one RCT [15]. Study populations were heterogeneous and included patients with acute cerebrovascular disease, intracerebral hemorrhage, neurocritical illness, and general hypertensive crises managed in ICU and hospital settings. Sample sizes ranged from 12 to 253 patients, with follow‐up durations varying from the infusion period to 30 days. Detailed study characteristics are presented in Table 1, while the studies contributing to each pooled outcome are summarized in Supporting Information S1: Table 4.

**Table 1:** Showing the study characteristics.

Study (year)	Design	Population (main)	Total *N*	Follow‐up duration	Key safety finding	Clinical Outcome
Finger et al. [11]	Retrospective	Neuro‐ICU (Mixed Stroke)	57	During infusion	Hypotension (SBP < 100): 15.8% versus 7.9%	% Time in Range: 79% versus 78% (NS)
Rosenfeldt et al. [12]	Retrospective	Acute Cerebrovascular Disease	119	Hospital stay	Hypotension (SBP < 90): 10.2% versus 3.3%	AKI: 37.2% versus 36.7% (NS)
Allison et al. [13]	Retrospective	Stroke (ICH & AIS)	210	Hospital stay	Hypotension (SBP ≤ 90): 7.1% versus 10.0% (*p* = 0.003)	Mortality: 15.7% versus 20.0% (NS)
Borrell‐Vega et al. [14]	Retrospective (Paired)	Neurocritical Care (Rescue)	12	During infusion	Rescue therapy data only	Infusion Duration: 32.4 versus 52.9 h
Johnson et al. [15]	Retrospective	General Hypertensive Crisis	182	Hospital stay	Hypotensive Excursion: 36.7% versus 33.0% (NS)	ICU LOS: 7.2 versus 6.15 days (NS)
Lv et al. [16]	RCT	General Hypertensive Crisis	253	12 h protocol	Hypotension (Overshoot): 0% versus 0.79%	Oral Transition: 96.8% versus 95.9% (NS)
Storey et al. [17]	Retrospective	General Hypertensive Crisis	156	Hospital stay	Hypotension: 36.6% versus 43.2% (NS)	ICU LOS: 3.3 versus 3.1 days (NS)
Armstrong et al. [18]	Retrospective (matched)	Mixed (Neuro, Aortic, Crisis)	200	During infusion +24 h	Hypotension (SBP < 90): 10% versus 17% (*p* = 0.093)	% Time in Range: 55.2% versus 59.0% (NS)
Saldana et al. [19]	Retrospective (matched)	Hemorrhagic stroke only	89	30‐day readmission	Rebound HTN: 75.9% versus 40% (*p* = 0.0017)	Mortality: 24.1% versus 26.7% (NS)

## 4 Meta‐Analysis Results

### 4.1 Time to Target SBP

**Figure 3:**
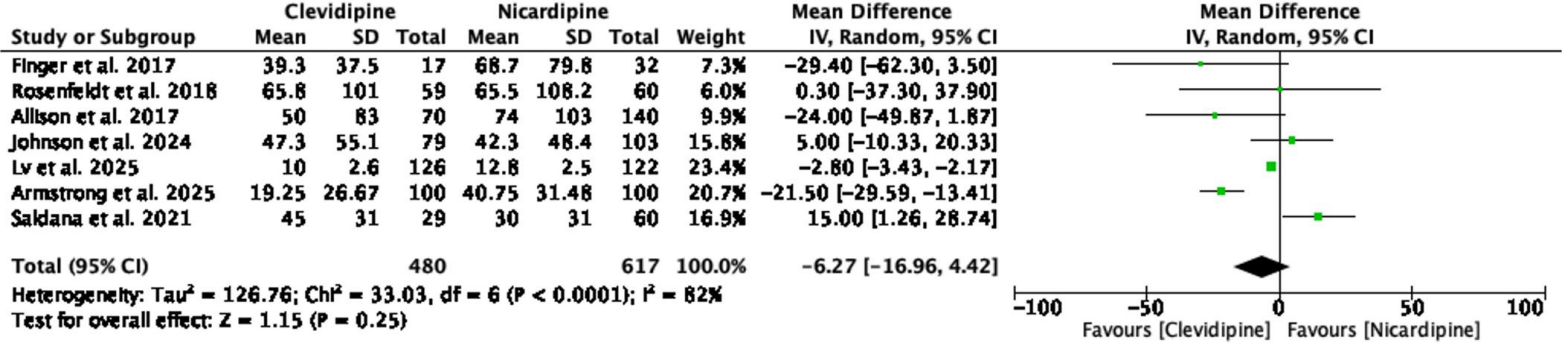
Shows the time to target SBP for both clevidipine and Nicardipine.

No statistically significant difference was observed between clevidipine and nicardipine in time to target SBP (MD −6.27 min, 95% CI −16.96 to 4.42; *I* ^2^ = 82%) (Figure 3). Considerable statistical heterogeneity was observed. To explore potential sources of heterogeneity, subgroup analyses were performed according to clinical setting (stroke/neurocritical care vs. general hypertensive crisis). Within both subgroups, clevidipine reached the target SBP significantly faster than nicardipine in the stroke/neurocritical care subgroup (MD −20.09 min, 95% CI −37.90 to −2.21; *I* ^2^ = 0%) and the general hypertensive crisis subgroup (MD −2.79 min, 95% CI −3.43 to −2.14; *I* ^2^ = 0%). The marked reduction in heterogeneity following subgroup analysis suggests that differences in patient populations contributed substantially to the observed heterogeneity (Supporting Information S1: Figure 10).

### 4.2 Percentage of Time Within Target SBP

**Figure 4:**
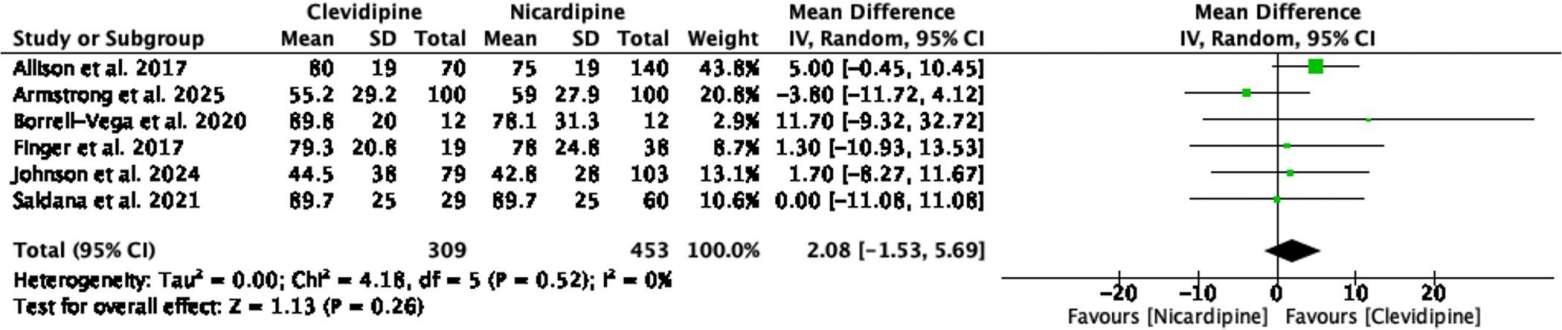
Shows the percentage age of time within target SBP both clevidipine and nicardipine.

There was no statistically significant difference between clevidipine and nicardipine in the percentage of time spent within the target SBP range (MD 2.08%, 95% CI −1.53 to 5.69; *I* ^2^ = 0%) (Figure 4). Subgroup analyses of stroke/neurocritical care and general hypertensive crisis populations similarly demonstrated no statistically significant differences (Supporting Information S1: Figure 11).

### 4.3 ICU Length of Stay (Days)

No statistically significant difference was observed in ICU length of stay between the two treatment groups (MD 0.81 days, 95% CI −0.42 to 2.05; low heterogeneity) (Supporting Information S1: Figure 1).

### 4.4 Hospital Length of Stay (Days)

Hospital length of stay did not differ significantly between clevidipine and nicardipine (MD 0.35 days, 95% CI −0.92 to 1.62; *I* ^2^ = 0%) (Supporting Information S1: Figure 2).

### 4.5 Infusion Drug Volume

Clevidipine was associated with a significantly lower infusion drug volume than nicardipine (MD −582.83 mL, 95% CI −860.49 to −305.18). Substantial statistical heterogeneity was observed (*I* ^2^ = 88%), likely reflecting differences in infusion concentrations, preparation protocols, and institutional prescribing practices across the included studies (Supporting Information S1: Figure 3).

### 4.6 Hypotension (Undefined)

No statistically significant difference was observed in the incidence of hypotension between the two groups (RR 0.90, 95% CI 0.72–1.12; *I* ^2^ = 0%) (Supporting Information S1: Figure 4).

### 4.7 Hypotension (≪90 SBP)

No statistically significant difference was observed in hypotension defined as SBP < 90 mmHg (RR 0.88, 95% CI 0.51–1.48), with low‐to‐moderate heterogeneity (Supporting Information S1: Figure 5).

### 4.8 Tachycardia

Similarly, tachycardia was found to be higher among nicardipine than clevidipine with RR: 1.21 (0.77, 1.90) with low heterogeneity among the studies (Supporting Information S1: Figure 6).

### 4.9 In Hospital Mortality

In‐hospital mortality did not differ significantly between the treatment groups (RR 0.83, 95% CI 0.51–1.36) (Supporting Information S1: Figure 7).

### 4.10 Acute Kidney Injury

No statistically significant difference was observed in the incidence of acute kidney injury between clevidipine and nicardipine (RR 1.00, 95% CI 0.63–1.58; *p* = 0.98) (Supporting Information S1: Figure 8).

### 4.11 Need for Rescue/Additional Antihypertensives

There was no statistically significant difference in the need for rescue or additional antihypertensive therapy between the treatment groups (RR 1.20, 95% CI 0.95–1.51) (Supporting Information S1: Figure 9).

## 5 Certainty of Evidence (GRADE Assessment)

The certainty of evidence was moderate across the evaluated outcomes. Moderate‐certainty evidence indicated no statistically significant difference between clevidipine and nicardipine in time to target SBP (MD − 6.27; 95% CI −16.96 to 4.42), percentage of time within the target SBP range (MD 2.08; 95% CI −1.53 to 5.69), ICU length of stay (MD 0.81; 95% CI −0.42 to 2.05), hospital length of stay (MD 0.35; 95% CI −0.92 to 1.62), hypotension, tachycardia, acute kidney injury, in‐hospital mortality, and the need for rescue/additional antihypertensive therapy. Clevidipine was associated with a significantly lower infusion drug volume (MD −582.83; 95% CI −860.49 to −305.18), supported by moderate‐certainty evidence (Supporting Information S1: Table 3).

## 6 Discussion

This systematic review compares the use of IV clevidipine and nicardipine for the rapid control of SBP in acute care settings. This systematic review includes nine studies that compared clevidipine with nicardipine in patients with hypertensive emergencies, including neurocritical care patients. Both drugs showed comparable efficacy in achieving the target SBP and maintaining blood pressure within the desired range [13, 15, 17]. Although the overall pooled analysis showed no significant difference in time to target SBP, subgroup analyses according to clinical setting demonstrated that clevidipine achieved the target SBP significantly faster in both the stroke/neurocritical care and general hypertensive crisis subgroups, with a marked reduction in statistical heterogeneity. No significant differences were observed in terms of hypotension, tachycardia, acute kidney injury, in‐hospital mortality, or hospital length of stay [18]. However, nicardipine was associated with significantly higher infusion drug volumes [25]. The finding of higher infusion drug volume with nicardipine was associated with significant heterogeneity. Collectively, these findings suggest that both agents are effective and safe. Practical considerations may influence agent selection in specific clinical contexts.

### 6.1 Efficacy Outcomes and Blood Pressure Control

Time to target SBP did not significantly differ between clevidipine and nicardipine [25]. The pharmacologic profile of clevidipine suggests that it has a theoretical advantage, but this was not observed in this study. Clevidipine is an ultra–short‐acting DHP CCB [26, 27]. It is rapidly metabolized by plasma esterases that allow its near‐immediate titration and rapid offset. In contrast, nicardipine has a longer effective half‐life and a more gradual offset profile [28, 29]. While this pharmacokinetic distinction might favor faster SBP control with clevidipine, the pooled findings did not consistently demonstrate this superiority. Several factors may explain this finding. First, there was substantial statistical heterogeneity in the time‐to‐target SBP analysis [30]. Subgroup analyses according to clinical setting (stroke/neurocritical care vs. general hypertensive crisis) markedly reduced heterogeneity, suggesting that differences in patient populations contributed to the observed variability. Second, the included studies encompassed patients with different clinical conditions, such as patients with ischemic stroke, intracerebral hemorrhage, neurocritical illness, and general hypertensive crises. These differences in study populations may have contributed to the observed heterogeneity. Third, the included studies used different SBP goals and urgency of reduction. In addition, infusion protocols, titration intervals, and monitoring intensity varied across institutions, which may have further contributed to the observed heterogeneity. In many settings, practical titration delays, nursing workflow, and protocol barriers may attenuate the theoretical speed advantage of clevidipine. Thus, real‐world implementation likely narrows pharmacologic differences between the agents.

The percentage of time within target SBP also did not significantly differ between groups, and it demonstrated low heterogeneity. This suggests consistent findings across these studies. Maintenance of blood pressure stability may be as clinically important as rapid reduction, particularly in cases of acute cerebrovascular and cardiovascular diseases [31, 32, 33, 34]. In these cases, excessive variability in BP has been associated with worse outcomes [35]. Although a slightly greater percentage of time within the target SBP range was observed with nicardipine, the difference was not statistically significant [36]. This may reflect the longer duration of action and steady hemodynamic profile of nicardipine. However, given the lack of statistical significance, both agents appear to be similarly effective in maintaining target SBP once it is achieved.

### 6.2 Safety Profile and Adverse Events

Safety outcomes were similarly comparable between the two agents. The incidence of hypotension did not differ significantly between the two agents. Overshoot hypotension may cause many complications therefore, it is clinically very important [37, 38, 39, 40]. Although clevidipine's rapid titratability theoretically reduces the risk of hypotensive overshoot but this advantage was not consistently observed in different studies [41]. Variation in hypotension definitions (SBP < 90 mmHg vs. < 100 mmHg) and monitoring strategies may have contributed to outcome variability.

The rates of tachycardia were also similar for both agents. As both agents are DHP CCB, reflex sympathetic activation and mild heart rate elevation are expected pharmacodynamic effects [42]. The absence of a significant difference suggests the comparable hemodynamic tolerability of the two agents. Importantly, there were no significant differences in acute kidney injury or in‐hospital mortality for both agents. Mortality in cases of hypertensive emergencies and acute stroke is largely driven by the severity of underlying disease [43]. It is not mainly driven by the specific IV antihypertensive agents used [44]. The included studies were likely underpowered to detect modest differences in hard clinical outcomes, and thus the absence of statistical significance should not be interpreted as definitive equivalence. Nevertheless, current evidence does not suggest superiority of one agent over the other in terms of survival or major organ‐related complications.

### 6.3 Infusion Drug Volume: Practical and Clinical Implications

One of the most notable findings of this analysis was the significantly higher infusion drug volume associated with nicardipine. This difference likely reflects the formulation characteristics and concentration requirements. Nicardipine is commonly administered in larger‐volume dilutions for continuous infusion, whereas clevidipine is formulated as a lipid emulsion that allows more concentrated delivery [45]. In neurocritical and critically ill populations, cumulative fluid balance may influence cerebral edema, pulmonary status, and overall ICU management [44]. Therefore, higher infusion volumes may have logistical and theoretical physiologic implications [46]. However, substantial statistical heterogeneity was observed for this outcome, limiting the certainty of the pooled estimate. This heterogeneity may reflect differences in institutional drug preparation protocols, infusion concentrations, dosing strategies, and local prescribing practices across the included studies. Although the amount of infusion fluid used may not always determine medication choice, it is a practical factor that should be considered, especially for patients who are sensitive to volume changes.

From a clinical perspective, the findings support the use of either clevidipine or nicardipine for acute SBP control in hypertensive emergencies and neurocritical settings. Both of the agents have comparable time to target SBP, and both of them demonstrated similar safety profiles. Therefore, the agent selection may depend on institutional familiarity, the cost consideration of the drug, and specific patient factors. Clevidipine may be efficient in cases that require extremely rapid titration and precise short‐term adjustments. Whereas, nicardipine may offer stable maintenance of BP once the target blood pressure is achieved. Importantly, no evidence suggests that the choice of agent significantly alters ICU or hospital length of stay. The variation in the number of studies contributing to each pooled analysis reflects differences in outcome reporting across the included studies rather than selective study inclusion.

This meta‐analysis has several limitations. Most of the included studies were retrospective cohort studies, with only one RCT, which increases the potential for selection bias, residual confounding, and limits the overall certainty of the evidence. There was substantial clinical heterogeneity, as the included studies enrolled diverse patient populations, including patients with stroke, intracerebral hemorrhage, neurocritical illness, and general hypertensive emergencies, with varying blood pressure targets, antihypertensive titration protocols, monitoring intensity, and outcome definitions. Although we performed subgroup analyses according to clinical setting (stroke/neurocritical care vs. general hypertensive crisis), which substantially reduced statistical heterogeneity for the primary outcomes, we were unable to perform additional subgroup analyses or meta‐regression based on blood pressure targets, dosing protocols, monitoring intensity, or study design because of the limited number of studies and inconsistent reporting of these variables. Significant statistical heterogeneity remained for some outcomes, particularly time to target SBP and infusion drug volume, which may have reduced the precision of the pooled estimates. In addition, the analysis may have been underpowered to detect differences in major clinical outcomes such as mortality and acute kidney injury. Follow‐up durations were generally short, limiting the assessment of long‐term clinical outcomes. Finally, factors such as drug cost, institutional treatment protocols, and medication availability were not consistently reported across studies, which may limit the generalizability of our findings.

## 7 Conclusion

In conclusion, the available evidence suggests that clevidipine and nicardipine provide comparable efficacy and safety for the management of hypertensive emergencies in acute care settings. Although clevidipine has pharmacokinetic properties that theoretically favor more rapid blood pressure control, these advantages did not consistently translate into superior clinical outcomes. Nicardipine was associated with higher infusion drug volumes; however, the clinical significance of this finding remains uncertain. Given that the available evidence is derived predominantly from observational studies with clinical heterogeneity, larger, high‐quality RCTs are needed to confirm these findings and better define the optimal antihypertensive agent in different clinical settings.

## Author Contribution

Conceptualization: Tahir Ullah, Hamed Khan, Anfal Khan. Data Curation: Tahir Ullah, Hamza Nasir. Formal Analysis: Anfal Khan, Kashif Yousaf. Funding Acquisition: None. Investigation: Abdul Qayum Khan, Muhammad Zaib. Methodology: Hamed Khan, Aleena Amir Malik. Project Administration: Anfal Khan, Aieman Naeem. Resources: Muhammad Zaib. Software: Kashif Yousaf. Supervision: Anfal Khan, Kashif Yousaf. Validation: Aleena Amir Malik, Abdul Qayum Khan, Syed Asad Ullah Agha, Aieman Naeem. Visualization: Kashif Yousaf, Hamza Nasir. Writing−Original Draft: Tahir Ullah, Hamed Khan, Hamza Nasir. Writing–Review and Editing: Anfal Khan, Aleena Amir Malik, Kashif Yousaf, Syed Asad Ullah Agha, Aieman Naeem. Graphical Abstract: Aieman Naeem.

## Funding

The authors have nothing to report.

## Ethics Statement

This study followed PRISMA guidelines and involved no direct human or animal subjects.

## Conflicts of Interest

The authors declare no conflicts of interest.

## Supporting information


Supporting File

## Data Availability

The data that support the findings of this study are available in the manuscript and the Supporting Information File.
