# Supplementary material for: Comparative Efficacy and Safety of Clevidipine Versus Nicardipine in Hypertensive Emergencies: A Systematic Review and Meta‐Analysis
Source: Clin Cardiol. 2026 Jul 16;49(7):e70419. doi: 10.1002/clc.70419 (PMC13373942; doi:10.1002/clc.70419)

**Supplementary Appendix**

**Table 1: Search strategy**

| **Databases** | **Search strategy** | **Number of articles retrieved** |
| --- | --- | --- |
| **Pubmed** | **(("Hypertension"[Mesh]**  **OR hypertension[All Fields]**  **OR hypertensive[All Fields]**  **OR "hypertensive emergency"[All Fields]**  **OR "hypertensive emergencies"[All Fields]**  **OR "hypertensive crisis"[All Fields]**  **OR "hypertensive crises"[All Fields])**  **AND**  **(clevidipine[All Fields]**  **OR nicardipine[All Fields]))** | **1248** |
| **Cochrane library** | **("hypertension" OR "hypertensive emergency" OR "hypertensive emergencies" OR**  **"hypertensive crisis" OR "hypertensive crises")**  **AND**  **(clevidipine OR nicardipine)** | **378** |
| **ClinicalTrials.gov** | (hypertension OR hypertensive crisis OR hypertensive emergency) AND (clevidipine OR nicardipine) | **03** |

**Table 2: Newcastle-Ottawa Scale (NOS)**

| Study  ID | SELECTION | | | | COMPARIBILITY | | OUTCOME | | | Total |
| --- | --- | --- | --- | --- | --- | --- | --- | --- | --- | --- |
|  | S1 | S2 | S3 | S4 | C1 | C2 | O1 | O2 | O3 |  |
| Allison2017 | * | * | * | - | * | - | * | * | * | 7* |
| Armstrong 2025 | * | * | * | - | * | - | * | * | * | 7* |
| Borrell‑Vega 2020 | - | * | * | - | * | - | * | * | - | 5* |
| Johson 2024 | * | * | * | - | * | - | * | * | * | 7* |
| Finger 2016 | * | * | * | - | * | * | * | * | * | 8* |
| Rosenfeldt 2018 | * | * | * | - | * | * | * | * | * | 8* |
| Saldana 2021 | * | * | * | - | * | * | * | * | * | 8* |
| Storey2024 | * | * | * | _ | * | - | * | * | * | 7* |

**Supplementary Table 3: GRADE Summary of Findings for Clevidipine vs Nicardipine**

| **Outcome** | **No. of Studies** | **Study Design** | **Risk of Bias** | **Inconsistency** | **Indirectness** | **Imprecision** | **Other Considerations** | **No. of Patients (Clevidipine/Nicardipine)** | **Relative/Absolute Effect** | **Overall Certainty of Evidence (GRADE)** |
| --- | --- | --- | --- | --- | --- | --- | --- | --- | --- | --- |
| Time to target SBP | 7 | 1 RCT + 6 observational studies | Not serious | Seriousᵃ | Not serious | Not serious | None | 480 / 617 | MD 6.27 minutes lower (95% CI 16.96 lower to 4.42 higher) | ⨁⨁⨁◯ Moderate |
| Percentage of time within target SBP | 6 | Observational studies | Not serious | Not serious | Not serious | Not serious | None | 309 / 453 | MD 2.08% higher (95% CI 1.53 lower to 5.69 higher) | ⨁⨁⨁◯ Moderate |
| ICU length of stay | 4 | Observational studies | Not serious | Not serious | Not serious | Not serious | None | 249 / 297 | MD 0.81 days higher (95% CI 0.42 lower to 2.05 higher) | ⨁⨁⨁◯ Moderate |
| Hospital length of stay | 4 | Observational studies | Not serious | Not serious | Not serious | Not serious | None | 240 / 334 | MD 0.35 days higher (95% CI 0.92 lower to 1.62 higher) | ⨁⨁⨁◯ Moderate |
| Infusion drug volume | 5 | 1 RCT + 4 observational studies | Not serious | Seriousᵃ | Not serious | Not serious | None | 336 / 401 | MD 582.83 mL lower (95% CI 860.49 lower to 305.18 lower) | ⨁⨁⨁◯ Moderate |
| Hypotension (Undefined) | 8 | 1 RCT + 7 observational studies | Not serious | Not serious | Not serious | Not serious | None | 577 / 713 | RR 0.90 (95% CI 0.72–1.12) | ⨁⨁⨁◯ Moderate |
| Hypotension (<90 mmHg) | 4 | Observational studies | Not serious | Not serious | Not serious | Not serious | None | 258 / 360 | RR 0.88 (95% CI 0.52–1.48) | ⨁⨁⨁◯ Moderate |
| Tachycardia | 5 | 1 RCT + 4 observational studies | Not serious | Not serious | Not serious | Not serious | None | 313 / 387 | RR 1.21 (95% CI 0.77–1.90) | ⨁⨁⨁◯ Moderate |
| In-hospital mortality | 2 | Observational studies | Not serious | Not serious | Not serious | Not serious | None | 99 / 200 | RR 0.83 (95% CI 0.51–1.36) | ⨁⨁⨁◯ Moderate |
| Acute kidney injury | 2 | Observational studies | Not serious | Not serious | Not serious | Not serious | None | 88 / 120 | RR 1.00 (95% CI 0.63–1.58) | ⨁⨁⨁◯ Moderate |
| Need for rescue/additional antihypertensives | 7 | 1 RCT + 6 observational studies | Not serious | Not serious | Not serious | Not serious | None | 297 / 441 | RR 1.20 (95% CI 0.95–1.51) | ⨁⨁⨁◯ Moderate |

### **Footnotes**

ᵃ Downgraded one level because of substantial statistical heterogeneity (I² >75%).

### **Supplementary Table 4. Studies Contributing to Each Outcome**

| **Outcome** | **No. of Studies** | **Studies Contributing (First Author, Year)** |
| --- | --- | --- |
| Time to target SBP | 7 | Finger et al., 2017; Rosenfeldt et al., 2018; Allison et al., 2017; Johnson et al., 2024; **Lv et al., 2026**; Armstrong et al., 2025; Saldana et al., 2021 |
| Percentage of time within target SBP range | 6 | Allison et al., 2017; Armstrong et al., 2025; Borrell-Vega et al., 2020; Finger et al., 2017; Johnson et al., 2024; Saldana et al., 2021 |
| ICU length of stay | 4 | Storey et al., 2024; Rosenfeldt et al., 2018; Johnson et al., 2024; Saldana et al., 2021 |
| Hospital length of stay | 4 | Allison et al., 2017; Rosenfeldt et al., 2018; Saldana et al., 2021; Storey et al., 2024 |
| Infusion drug volume | 5 | Finger et al., 2017; Johnson et al., 2024; **Lv et al., 2026**; Saldana et al., 2021; Storey et al., 2024 |
| Hypotension (study-defined) | 8 | Allison et al., 2017; Armstrong et al., 2025; Finger et al., 2017; Johnson et al., 2024; **Lv et al., 2026**; Rosenfeldt et al., 2018; Saldana et al., 2021; Storey et al., 2024 |
| Hypotension (SBP <90 mmHg) | 4 | Allison et al., 2017; Armstrong et al., 2025; Rosenfeldt et al., 2018; Saldana et al., 2021 |
| Tachycardia | 5 | Finger et al., 2017; Johnson et al., 2024; **Lv et al., 2026**; Rosenfeldt et al., 2018; Saldana et al., 2021 |
| In-hospital mortality | 2 | Allison et al., 2017; Saldana et al., 2021 |
| Acute kidney injury | 2 | Rosenfeldt et al., 2018; Saldana et al., 2021 |
| Need for rescue/additional antihypertensives | **7 reported; 5 contributed quantitative data** | Allison et al., 2017; Armstrong et al., 2025; Finger et al., 2017; Johnson et al., 2024; Saldana et al., 2021 |

**Footnote:**

The number of studies contributing to each pooled analysis varied because not all included studies reported every outcome. Lv et al. (2026) was the only randomized controlled trial included in this review; all remaining studies were retrospective cohort studies.

**Forest Plots of Outcomes**

**Figure 1 Supplementary:** ICU length of stay in days


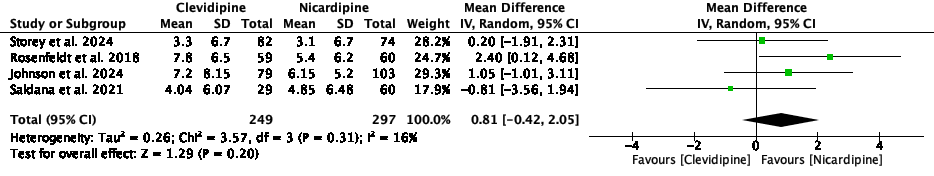


**Figure 2 Supplementary:** Hospital length of stay in days


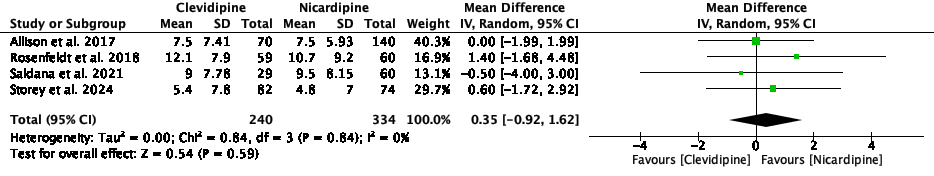


**Figure 3 Supplementary:** Infusion Drug Volume


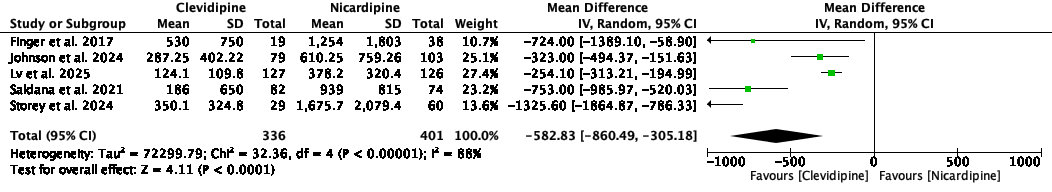


**Figure 4 Supplementary:**Hypotension (Undefined)


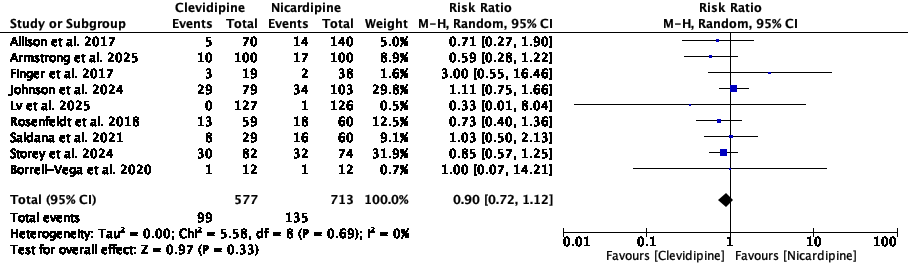


**Figure 5 Supplementary:**Hypotension (<90 SBP)


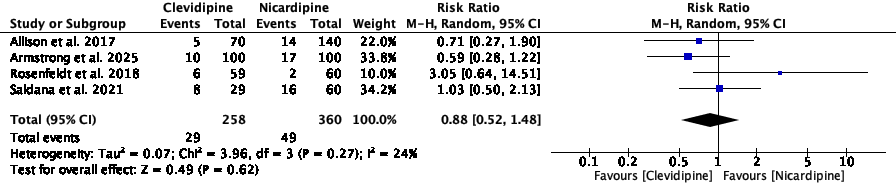


**Figure 6 Supplementary:**Tachycardia


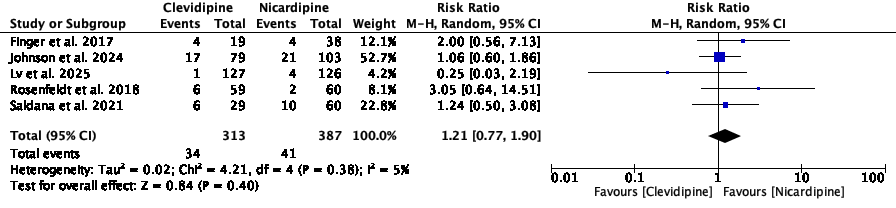


**Figure 7 Supplementary:**In hospital Mortality


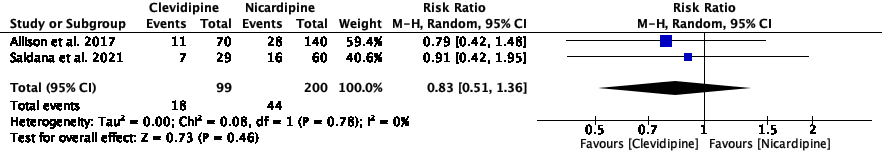


**Figure 8 Supplementary:**Acute kidney injury


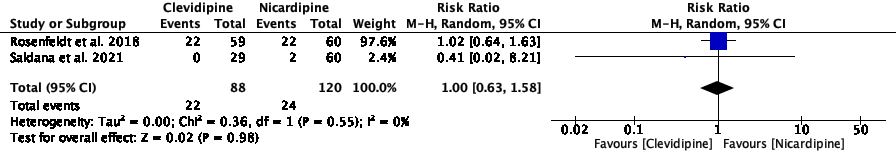


**Figure 9 Supplementary:**Need for rescue/ additional antihypertensives


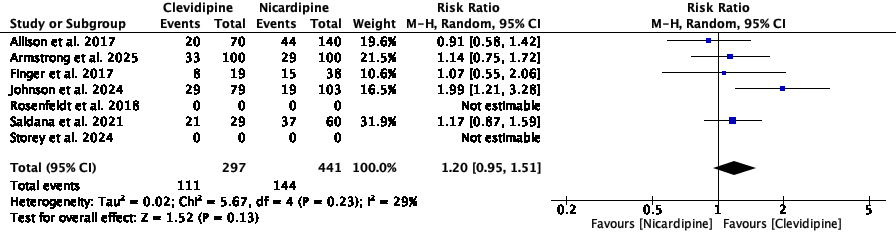


**Figure 10 Supplementary: Time to target SBP Subgroup analyzed by Stroke/ Neurocritical and General** hypertensive crisis population


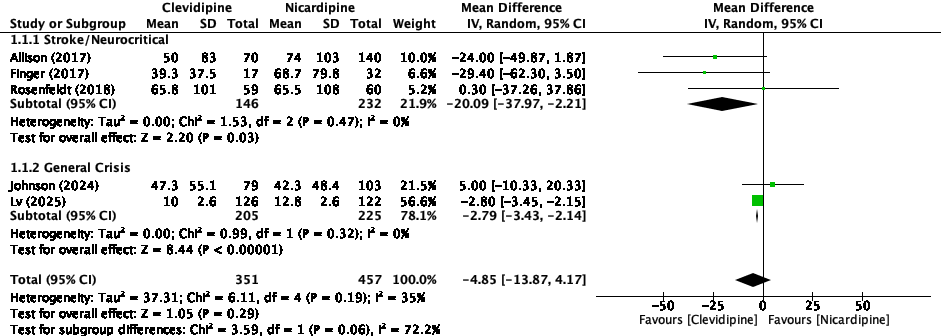


**Figure 11 Supplementary:** Percentage of Time within Target SBP Range: Subgroup Analysis by Stroke/Neurocritical Care and General Hypertensive Crisis Populations


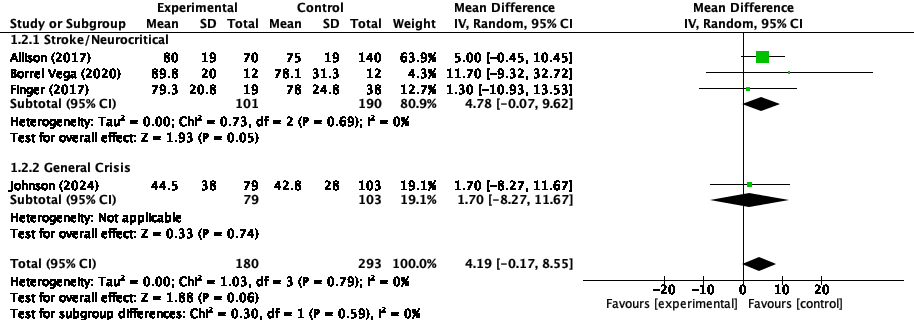

Supplement: Supplementary file 1 — Supporting File [file CLC-49-e70419-s001.docx]
